# Cerebral Abscess

**Published:** 1908-07-25

**Authors:** Percy Sargent

**Affiliations:** Assistant Surgeon to St. Thomas's Hospital and to the National Hospital for the Paralysed and Epileptic, Queen Square.


### Hospital Clinics.

### CEREBRAL ABSCESS.

By PERCY SARGENT, F.R.C.S., Assistant Surgeon to St. Thomas's Hospital and to the National Hospital for the Paralysed and Epileptic, Queen Square.

Abstract of a Lecture delivered at the National Hospital.

As the subject of my lecture does not lend itself to clinical demonstration, I must confine myself to pathological specimens and accounts of cases to illustrate my remarks. Cerebral abscess is by no means a common disease. During seven years (1899-1905) only 42 cases were admitted into St. Thomas's Hospital. During the same period 780 cases of suppurative otitis media were admitted to the wards, and 2,250 were treated in the out-patient department, so that even of those suffering from what is the most frequent cause of cerebral abscess only just over 1 per cent. developed that complication. When we consider the enormous number of patients with otorrhœa who go untreated altogether it is apparent how rarely this condition results, even from its commonest cause.

*Age and Sex.*—Cerebral abscess is much more frequent in males than in females; in the series I am considering there are 28 males and 14 females. In point of age most of the cases occur between 10 and 30. Thus, of these 42 cases nine were under 10 years of age, 27 between 10 and 30, and only six over 30.

### Ætiology.

As I have already said, suppurative disease of the middle ear and of the temporal bone is by far the commonest cause, no fewer than 38 of the 42 cases being of this nature.

Of the rest, injury is responsible for a certain number of cases. Sometimes there has been a penetrating wound, sometimes the primary cause is necrosis of some part of the skull consequent upon an injury. In other cases, again, there has been no external wound at all. Of this the following cases are good illustrations. A man 35 years of age had, six weeks before admission, received a blow upon the head which rendered him unconscious. Shortly afterwards he began to have fits, and some operation was performed, but the symptoms persisted, and he came under the care of Mr. C. A. Ballance, who operated and drained an abscess. In another instance a man of 37 years of age was struck over the right eye two months before coming to hospital. He was knocked down, but did not lose consciousness; five or six weeks after the injury he commenced to vomit and to be drowsy, but he continued at work until six days before admission, when he was found to have weakness of the left arm and leg, and left facial paralysis; the pupils were small and sluggish, and the pulse rate only 44. He died from respiratory failure two days after admission. At the autopsy there was found no meningitis, fracture, or sinus thrombosis, but there were four separate and distinct abscesses in the brain. The largest was the size of a walnut, and was situated in the anterior part of the right temporal lobe; the others were smaller and were situated one in the right precentral convolution, one in the right angular gyrus, and the third in the left quadrate gyrus.

Most of the traumatic cases, however, are due to necrosis following compound fracture, and the pathology of these is similar to that of the cases that follow suppurative middle-ear disease.

Other occasional causes of cerebral abscess are necrosis of the bones of the nose, pyæmia and tuberculosis; the tuberculous cases form rather a class by themselves, for it is not common for a caseous mass in the brain to soften and form an abscess; they most often run the course of a cerebral tumour. Abscesses of the brain complicating pyæmia, arising from causes other than thrombosis of the lateral sinus, were rare even in the days when pyæmia was a common disease, and it is probable that those which complicate lateral sinus pyæmia are really extensions from a common focus, rather than truly metastatic in nature.

Bronchiectasis is an occasional primary source of infection for metastatic abscess of the brain. In this connection one of the cases in the St. Thomas's series is of interest. The patient was a man, 30 years of age, who had suffered from some pulmonary mischief when 17 years old, but in the interval had been in fairly good health until 15 months before admission, when he began to have shivering fits. Five days before he came to hospital he developed tingling and loss of power in the right arm and leg, accompanied by spasmodic movements; he had also intense headache; the face was unaffected, nor was there any impairment of sensation anywhere; there was no aphasia, and no optic neuritis; the fingers were clubbed, and the lower lobe of the left lung presented physical signs of cavitation. On the day after admission he had a Jacksonian fit, and a second similar fit occurred on the following day; after each fit the weakness was more marked. On the eighth day after admission the left Rolandic area was exposed, and a foul abscess opened and drained. After temporary improvement he became unconscious, and two days later the wound was re-opened in order to secure better drainage. Within a fortnight he had improved greatly, and this improvement was maintained, so that in three months he left hospital with the wound healed, and almost well, the only remaining symptom being slight weakness of the right hand. He continued well for five months, when he developed a right-sided hemiplegia. He was re-admitted and pus was again evacuated from the same situation, but death ensued four days later. At the autopsy the abscess cavity was found to extend into the lateral ventricle, which was full of pus; there was a second abscess the size of a walnut above that which had been drained; a little basal meningitis also was present; the lower lobe of the left lung was riddled with bronchiectatic cavities.

### Pathology.

I do not know of any large series of cases in which the bacteriology of cerebral abscess has been fully worked out, and it is impossible to give a correct idea of the relative frequency of the organisms concerned. Amongst the commoner ones are undoubtedly the staphylococcus pyogenes aureus and streptococcus pyogenes; others that have been less commonly found are the staphylococcus citreus, staphylococcus albus, pneumococcus, and bacillus pyocyaneus. Many of these abscesses, however, are sterile to cultivation, although organisms may be seen in stained films, and in this respect are comparable to certain cases of appendix abscess. The appearance of the pus varies. It is usually described as being thick, and greenish in colour; but it is sometimes, especially in the more acute cases, thin and watery, and of a brownish colour; sometimes the cavity contains gas with the pus. The odour is often extremely offensive, but it is quite valueless as an indication either of the particular organism concerned, or of virulence, just as is the case with appendix abscesses. The capsule is very variable. Some abscesses possess no capsule, their walls being composed merely of ragged necrotic brain tissue, but others have a wall consisting of a considerable thickness of fibrous tissue, and distinct from the surrounding brain substance. Such a capsule may be more or less uniform, or may present thin spots through which extension to surrounding parts of the brain may take place. In other cases without any obvious change in the capsule itself the surrounding cerebral substance may become rapidly softened and œdematous. In other instances, again, the contents of the abscess cavity may become inspissated. A thick capsule is evidence of chronicity, but a chronic abscess does not necessarily possess a thick capsule. Mr. Ballance has reported a case of cerebellar abscess of eight months' duration in which there was no evidence whatever of a capsule.

*Mode of Infection.*—The organisms may gain entrance by direct inoculation, as by a penetrating wound, a gunshot injury, or by fragments of bone driven into the brain in a compound fracture. Hæmic infection has already been referred to. In the cases complicating disease of bone the infection enters the cranial cavity either directly through the carious bone, through a suture, or through a foramen transmitting a small vessel. The resistance of the dura varies very greatly, and in some cases the infection remains extra-dural for a long while. During this period the membranes are becoming adherent, so that by the time the dura is perforated the general subdural space has become shut off. The cortex, on account of its vascularity, is more resistant to the spread of infection than is the white matter, so that when once the cortex has been traversed, the resulting abscess is apt to spread out in the white matter in a flask-shaped manner.

It is not very rare to find multiple brain abscesses. These may be formed by direct extension from one to another, or from a common primary focus, or they may be pyæmic in nature. In the forty-two cases which I have tabulated there are six examples of multiplicity.

The traumatic cases and those arising from disease in connection with the nose, are commonest in the frontal lobes; in the aural cases either the temporal lobe or the cerebellum may be affected. It has been stated that cerebral abscess is four times more frequent than cerebellar, but MacEwen throws doubt upon this statement, and Ballance asserts that the cerebellum is the more frequent situation. In the thirty-eight cases of the series I have quoted, which resulted from middle-ear disease, the abscess was situated twenty-two times in the temporal lobe, thirteen times in the cerebellum and in the remaining three both these regions were involved. Rarer situations are the pons and medulla.

### Clinical Features.

An abscess may develop without any obvious exciting cause, but occasionally a head injury in a person suffering from middle-ear disease has been the determining factor. Again, operation sometimes precedes the onset of symptoms. Thus in my series one patient had four days previously had a granulation polypus removed from the ear; in another the grafting stage of the complete mastoid operation had been done six days previously. The third was a case of my own; that of a child ten years of age, who had been operated upon for adenoids fifteen days before admission to hospital. At the time of the operation the child was apparently quite healthy, but a week later pain occurred in the right ear, and shortly afterwards otorrhœa and vomiting. When admitted she was unconscious and hemiplegic on the left side. The deep reflexes were absent on the left side, and diminished on the right; the right pupil was widely dilated and immovable; there was slight divergent squint. The optic discs were normal. There was slight aural discharge but no œdema over the mastoid; the temperature was 102° F. The complete mastoid operation was done at once, and on removing the necrosed tegmen tympani about an ounce and a half of pus escaped from a cavity within the temporal lobe. On the following day the child was conscious, and could move the limbs fairly strongly, but on the fifth day a hernia cerebri appeared and she gradually became worse. Death took place on the eleventh day. At the autopsy basal meningitis was found, and the whole of the temporal lobe was occupied by a large abscess. There was also widespread septic broncho-pneumonia.

*Diagnosis.*—The following factors may create difficulty in the diagnosis of a cerebral abscess complicating middle-ear disease. Firstly, suppurative otitis media may be present and give rise to intracranial suppuration without any perforation of the drum, or discharge from the meatus. Secondly, acute middle-ear disease may of itself give rise to vomiting, rigors, facial palsy, and even optic neuritis. Thirdly, some other intracranial complication may be present at the same time and obscure the symptoms of the cerebral abscess. Thus, in the thirty-eight cases referred to, sinus thrombosis was present in six, extra-dural abscesses in three, and meningitis in ten.

*Course of the Disease.*—The course which a cerebral abscess may run, varies greatly. Some cases are so acute as to defy diagnosis, others so chronic as to be mistaken for cerebral tumour. In others, again, an abscess may exist for some time and cause no obvious symptoms until some change, such as rupture into the ventricle or into the subdural space occurs, bringing about a rapidly fatal termination. Some abscesses are even discovered accidentally during the course of a mastoid operation. Such cases, however, are uncommon, and ordinarily a cerebral abscess shows the three stages described by MacEwen. In the stage of *onset* there are headaches, vomiting, quickened pulse, raised temperature, and rigors. In the *second* stage there is less vomiting, the pulse is often slow, and the temperature may fall; the patient is drowsy and exhibits a characteristic vacant expression; constipation is a marked feature, and polyuria and even glycosuria are not uncommon. The *terminal* stage is characterised by slow coma, or death from respiratory failure, whilst symptoms of meningitis frequently manifest themselves.

### Individual Symptoms.

When the abscess is fully developed the symptoms present are referable, some to intra-cranial pressure, and some to toxæmia, whilst others are of localising value.

(*a*) *Pressure Symptoms.*—The *headache*, which is often extremely severe, may be general or localised to one particular part of the head; occasionally the situation of the pain is of localising value. The *vomiting* is of the usual cerebral type, that is to say unrelated to food and unaccompanied by nausea. *Optic neuritis* is generally unequal on the two sides, and slight in degree; it interferes but little with vision, and is rarely succeeded by optic atrophy; it may be absent altogether, as in four out of fifteen cases of temporal abscess, and in three out of thirteen cerebellar abscesses. In the remaining eleven temporal cases the optic neuritis was unilateral in three, bilateral and equal in six, and bilateral but more intense on the side of the lesion in two. On the other hand, it must not be forgotten that optic neuritis is not infrequently present in complications of middle-ear disease other than cerebral abscess.

(*b*) *Toxic Symptoms.*—The temperature is often extremely misleading. It may be high and accompanied by rigors, but both these symptoms may also be due entirely to the middle-ear disease, or to some other intracranial complication. In mild infection or in very chronic cases, there may be no rise of temperature at all, and sometimes it remains subnormal, with occasional slight temporary elevations at fairly regular intervals. In spite of septic absorption the temperature may be normal or subnormal throughout, and the pulse very slow; in such cases the usual symptoms of septic absorption are masked by the accompanying intracranial pressure. Cushing has advanced the theory that the high blood pressure of cerebral hæmorrhage is a natural compensatory effect, in order to ward off the anæmia of the medullary centres which would otherwise occur in consequence of the increased intracranial pressure. It is possible that a similar mechanism may come into play in cases of cerebral abscess.

(*c*) *Localising Symptoms.*—I can only indicate very briefly the symptoms that would guide the surgeon in operating for cerebral abscess. There may be none at all, or on the other hand a large abscess, especially if surrounded by a large area of softening, may cause very wide-spread symptoms.

In abscess of the *temporal lobe* motor symptoms may be either of the cortical type or of the internal capsule type; in the latter hemi-anæsthesia may be present. Paralysis of the third nerve on the side of the lesion occurs fairly frequently. Aphasia is often present, and may be either motor or sensory. In two cases of my series word blindness was present. The mental condition is often peculiar, and has been stated to be almost characteristic of abscess in this part of the brain. The patient is listless and apathetic, but he can be roused; when asked a question he may make no reply, and appear to take no notice, but if given time, he will ultimately answer the question, and usually quite correctly. One must be careful not to mistake this condition for aphasia. Of the special senses, hearing is naturally often affected by the original ear disease. Anosmia is not infrequently present when the abscess is situated near the anterior end of the temporal lobe, and occasionally subjective sensations of smell have been noted.

In *cerebellar* cases the abscess may be situated in the interior of one lateral lobe, and may, or may not involve the dentate nucleus; it is very rare to find the vermis alone involved, although an abscess in the lateral lobe not infrequently extends into it. Again, the abscess is often situated at the extreme anterior edge of the lateral lobe, close to the diseased bone from which it originated. The symptoms, therefore, will vary according to the exact situation of the abscess. There may be the characteristic cerebellar gait and attitude, incoordination, nystagmus, and signs of pressure on the pyramidal tract. Facial paralysis is sometimes seen quite apart from the local effects of the middle-ear disease. In one case in my series the hypoglossal nerve was affected.

### Treatment.

The results of treatment are unfortunately disappointing. In a few recorded cases spontaneous evacuation through the roof of the tympanum has occurred, and recovery, temporary at least, has ensued, but these are to be regarded merely as curiosities; in some cases death has occurred months after an apparently successful operation. Of the thirty-eight cases quoted in which the abscess was of aural origin six recovered completely, but in two no operation was attempted, and in only twenty-two was the cavity drained. The mortality may therefore be put down approximately as 80 per cent.

With regard to exploratory puncture, there are several fallacies. The trocar used is nearly always much too small to allow of the thick pus making its way along it, and moreover the trocar very easily becomes blocked by brain tissue. Again, an abscess wall may be so thick and resistant as to be pushed bodily away by the trocar in the yielding brain substance. In many cases which come to the post-mortem room it is found that the exploratory puncture has narrowly missed an abscess. In a case of my own the track of the trocar was within one eighth of an inch of the abscess wall.

If possible, an abscess due to bone disease should be drained along the same track as that along which the infection has travelled. If the situation of an abscess due to mastoid disease is uncertain, either the temporal lobe or the cerebellum can be explored by removing a reasonably large area of bone behind the mastoid wound; for it need scarcely be said that in such a case the complete mastoid operation must first be performed. The fatal termination of many of the cases in which an abscess has been drained results from either a spreading encephalitis, extension into a ventricle, or diffuse meningitis. Such complications are made evident by the appearance of a hernia cerebri some days after the operation, and are usually due to imperfect drainage. In order to open and drain efficiently a cerebral abscess, a free incision with a scalpel should be made through the overlying brain substance; the finger inserted and a large drainage tube stitched in position. If the track becomes blocked by the falling together of the lips of the incision, it may be advisable to cut away some of the outer wall of the cavity in order to secure proper drainage. After the operation the cavity must be frequently washed out through the tube with some mild antiseptic solution, and the tube gradually shortened; if the brain bulges there should be no hesitation in freely re-opening the wound.

